# Force–conductance spectroscopy of a single-molecule reaction†
†Electronic supplementary information (ESI) available. See DOI: 10.1039/c8sc04830d


**DOI:** 10.1039/c8sc04830d

**Published:** 2019-01-25

**Authors:** Leopoldo Mejía, Ignacio Franco

**Affiliations:** a Department of Chemistry , University of Rochester , Rochester , New York 14627-0216 , USA . Email: ignacio.franco@rochester.edu; b Department of Physics , University of Rochester , Rochester , New York 14627-0216 , USA

## Abstract

We demonstrate how simultaneous measurements of conductance and force can be used to monitor the step-by-step progress of a mechanically-activated *cis*-to-*trans* isomerization single-molecule reaction, including events that cannot be distinguished using force or conductance alone. To do so, we simulated the force–conductance profile of cyclopropane oligomers connected to graphene nanoribbon electrodes that undergo a *cis*-to-*trans* isomerization during mechanical elongation. This was done using a combination of classical molecular dynamics simulation of the pulling using a reactive force field, and Landauer transport computations of the conductance with nonequilibrium Green's function methods. The isomerization events can be distinguished in both force and conductance profiles. However, the conductance profile during the mechanical elongation distinguishes between reaction intermediates that cannot be resolved using force. In turn, the force signals non-reactive deformations in the molecular backbone which are not visible in the conductance profile. These observations are shown to be robust to the choice of electrode and Hamiltonian model. The computations exemplify the potential of the integration of covalent mechanochemistry with molecular conductance to investigate chemical reactivity at the single-entity limit.

## Abstract 2

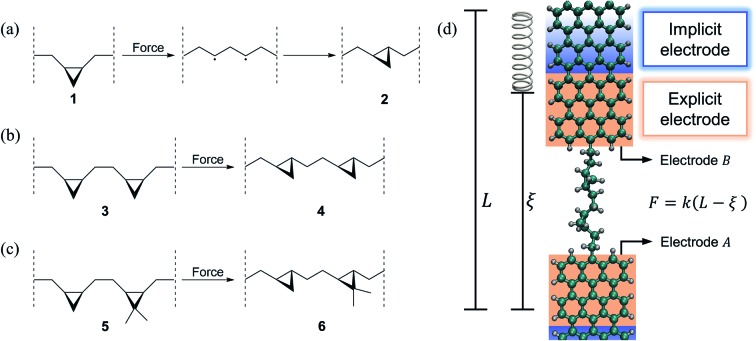
We demonstrate how simultaneous measurements of conductance and force can be used to monitor the step-by-step progress of a mechanically activated *cis*-to-*trans* isomerization single-molecule reaction, including events that cannot be distinguished using force or conductance alone.

## 1 Introduction

Controlling and monitoring single-molecule processes, and in particular, single-molecule reactions, has received considerable attention in recent years.^1^–^13^ The development of instruments and techniques that allow the manipulation of single-molecules, such as atomic force microscopy (AFM) and scanning tunneling microscopy (STM), has made it possible to trigger specific reaction pathways during single molecule-reactions by applying external stimuli.^1^,^4^,^5^,^7^,^11^,^13^

Among the possible external stimuli that can trigger a single-molecule reaction, mechanical forces have proven to be suitable to induce a wide variety of them including cyclizations,^14^ ring-openings,^15^–^23^ dissociations,^24^–^26^ isomerizations,^27^–^31^ electron-transfers^32^ and others.^25^,^26^,^33^–^41^ The application of mechanical forces to single-molecules effectively changes the potential energy surface^15^–^17^,^20^ and allows triggering of formally forbidden or sterically hindered reactions.^18^,^19^,^24^,^28^,^30^,^31^ These kinds of processes can be monitored by plotting the force exerted against molecular extension.^18^,^19^,^21^,^22^,^28^,^29^ The force–extension isotherms signal structural transitions or changes in mechanical elasticity.^42^,^43^ However, they are limited when it is desirable to distinguish subtle structural changes or events that occur at the same force.

A complementary observable to force that can be implemented in the same experimental setup is conductance.^44^–^46^ Conductance signals changes in the transport-determining molecular electronic energy levels. Therefore, it can reveal chemical processes that are not evident in the force profile. While some chemical transformations have been monitored using conductance alone,^2^,^4^,^5^,^32^,^47^ the combination of force and conductance as a general route for investigating chemical reactivity has not been explored before.

**Fig. 1:**
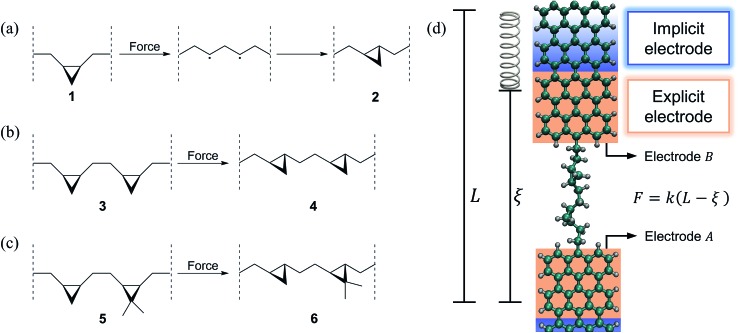
(a–c) Mechanically induced *cis*-to-*trans* single-molecule isomerization reactions and (d) schematic representation of the force–conductance spectroscopy simulation setup. We simulated the isomerization of a molecule with (a) a single, (b) two equivalent and (c) two non-equivalent substituted cyclopropane rings. The ring opening leads to the formation of diradical intermediates that allow molecular rearrangement as detailed in (a). These molecules are connected to graphene nanoribbon (GNR) electrodes and mechanically deformed as schematically shown in (d). During pulling, one GNR electrode is kept fixed while the other one is connected to a virtual spring of stiffness *k* = 0.28 N m^–1^, with equilibrium position *L* = *L*_0_ + *vt* moving at a constant speed of *v* = 10^–6^ Å fs^–1^. The deflection of the spring (*L* – *ξ*) measures the force exerted *F* = *k*(*L* – *ξ*) during the pulling, where *ξ* is the extended-molecule length. In the transport computations, the explicit GNR electrodes are connected to macroscopic implicit GNR electrodes.

Here we demonstrate how simultaneous force–conductance measurements can be used to monitor the step-by-step progress of mechanically activated single-molecule isomerization reactions, revealing molecular events that cannot be distinguished using force or conductance alone. Specifically, we simulated three exemplifying *cis*-to-*trans* isomerizations of cyclopropane-based systems, in the context of mechanically deformed graphene nanoribbon (GNR) junctions. These simulations complement the recent experimental progress in inducing *cis*-to-*trans* isomerization reactions in polymeric systems using AFMs.^28^,^29^ Fig. 1a–c show the simulated isomerization reactions including a system with a single (a), two equivalent (b) and two nonequivalent (c) cyclopropane-like rings. All isomerizations are preceded by a force-induced ring opening as depicted in Fig. 1a. These molecules are connected to GNR electrodes and mechanically deformed as schematically shown in Fig. 1d. As electrodes we chose GNRs, instead of Au, because they are conductive and the C–C bond between the GNR and the molecule can endure the relatively large tensile forces (∼2 nN) that are required to trigger these reactions,^48^,^49^ preventing the breaking of the junction. Electrodes similar to these have been successfully used before in experiments^2^,^47^ and simulations.^3^,^6^

These results illustrate the power of force–conductance measurements as a general platform for the development of highly discriminating multidimensional single-molecule spectroscopies,^44^,^46^ and represent new frontiers in the control of chemical processes at the single-molecule limit.

## 2 Methods

### 2.1 Force spectroscopy and pulling simulations

Force spectroscopy was modeled using classical molecular dynamics (MD) simulations performed in the NVT ensemble using LAMMPS.^50^ The temperature was fixed at 300 K by applying a Langevin thermostat^51^ (with 50 fs damping parameter) and the equations of motion were propagated using an integration time step of 0.5 fs. To capture bond breaking and forming we employed the reactive force field ReaxFF,^52^,^53^ which contains parameters for reactive interactions in hydrocarbons. ReaxFF provides accuracy similar to density functional theory (DFT) simulations but with the computational cost of classical force fields. To include the effects of the electrodes in the molecular dynamics, two 28-carbon fragments of an *armchair* graphene nanoribbon (GNR) (corresponding to the explicit electrode shown in Fig. 1d) were considered in the dynamics as part of the extended molecule. The molecule was connected to the GNRs through C–C bonds as shown in Fig. 1d. The molecular pulling was simulated by applying a force to the center of mass of the last carbon layer of electrode B, while electrode A was kept static by attaching the center of mass of its last carbon layer to a stiff isotropic harmonic potential. As shown in Fig. 1d, such a force is applied by connecting the center of mass of electrode B to a virtual harmonic spring with equilibrium position *L*. The virtual spring has an effective stiffness of *k* = 0.28 N m^–1^ along the pulling direction and is rigid in perpendicular directions. During pulling, the equilibrium position of the virtual spring *L* = *L*_0_ + *vt* is moved away from the molecule at a constant speed *v* = 10^–6^ Å fs^–1^ where *L*_0_ is the starting position. The deflection of the spring from its equilibrium position measures the force during the pulling *F* = *k*(*L* – *ξ*), where *ξ* is the distance between the two ends of the explicit electrodes (the extended molecular length). Since the simulated reactions are rare events in the dynamics, Replica Exchange Molecular Dynamics (REMD)^54^ as implemented in LAMMPS was used to enhance the sampling in the systems that contain more than one ring (Fig. 1b and c). For each of these reactions, seven replicas were simulated with temperatures of 280, 300, 330, 340, 380, 400 and 450 K and using an exchange attempt frequency of 5 ps^–1^. After the dynamics, the trajectories were processed to select the structures that belong to the ensemble of interest (300 K).

### 2.2 Transport computations

In the low bias limit, molecular conductance 
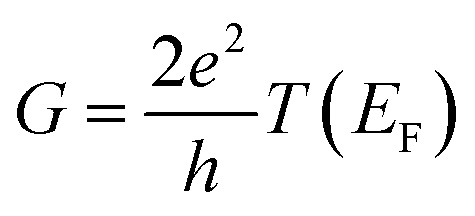
 is proportional to transmission at the Fermi energy *T*(*E*_F_).^55^ The quantity *T*(*E*_F_) was computed for ∼70 000 molecular snapshots encountered during the molecular dynamics and averaged in 0.01 Å bins along the *ξ* coordinate. To do so, we employed nonequilibrium Green's functions^56^ with two semiempirical Hamiltonians: Extended Hückel (EH)^57^,^58^ and Self-Consistent Charge Density Functional Tight-Binding (SCC-DFTB).^59^ Both methods capture the essential electronic structure of the system and enable the simulation of several thousand conformations at a reasonable computational cost as required for this analysis. In the EH method, while the density of states of the electrodes was considered in the wide band limit approximation,^60^ the atoms in the explicit electrode (see Fig. 1d) were employed in the computations to define the electrode–molecule couplings. The Fermi energy of the electrodes was taken to be at the center of the HOMO–LUMO gap of a 40 Å long GNR, yielding *E*_F_ = –10.78 eV. In turn, the DFTB transport computations were done with DFTB+^61^ using self-consistent charges, together with the “mio” Slater–Koster parameters.^59^ To fulfill the periodicity requirements of the electrodes' principal layers of DFTB+, the explicit electrodes included in the molecular dynamics were replaced by pristine GNRs. The closest part of the electrode (with respect to the molecule), corresponding to the explicit electrode, was included in the so-called molecular region. Another two equivalent fragments (per electrode) were appointed as the first and second principal layers. These SCC-DFTB simulations were carried out considering temperatures of 0 K and 300 K, without invoking the wide band limit approximation.

## 3 Results and discussion

**Fig. 2:**
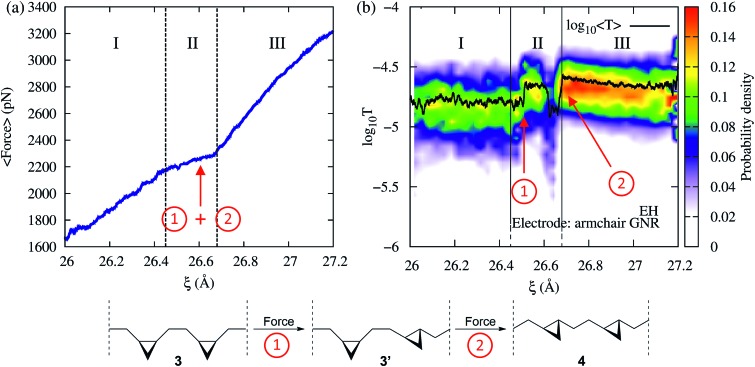
(a) Average force and (b) EH transmission distribution (color) and average (black line) as a function of the extended-molecule length, *ξ*, during the mechanically activated isomerization of the system with two equivalent cycles 3→4 (bottom panel). The force in region II signals the isomerization of both cycles. Note that, while in the force profile both isomerizations [circle containing 1] and [circle containing 2] are identified as a single event, the transmission profile clearly distinguishes between them.

Fig. 2 shows the force and conductance profiles during the mechanically activated *cis*-to-*trans* isomerization of the system with two equivalent cycles 3→4. The transmission was computed using the EH Hamiltonian and *armchair* GNR electrodes. Three regions denoted as I, II and III can be clearly distinguished in force and transmission profiles. Regions I and III are nonreactive regions, while in region II the isomerization of both cycles takes place. While the force profile indicates that there is mechanical deformation of the system in regions I and III, the stability of the conductance suggests that there are no significant transport-determining structural or conformational changes in these two regions. By contrast, the increase in the elasticity of the system in region II, resulting in a lower slope 
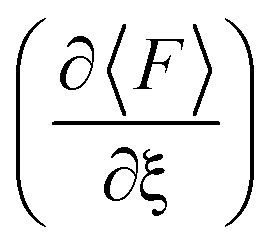
 in the *F*–*ξ* isotherm, is accompanied by a highly active transmission profile that signals conformational and structural changes, including the isomerization of both cycles. The isomerization of both cycles results in the emergence of a single approximate plateau in the force profile (Fig. 2a). The computed force of ∼2200 pN required for inducing the 3→4 reaction and the overall shape of the force profile are comparable to those observed in related experiments where a force of ∼1300 pN was required to induce the *cis*-to-*trans* isomerization of *gem*-difluorocyclopropane^29^ and *gem*-dichlorocyclopropane-containing^28^ polymers. Differences between theory and experiments arise because the systems are not identical, involving different linker groups to the pulling device, substituents in the cyclopropanes, and solvent environment.

Remarkably, although the force profile can signal that there is a reaction, only the conductance profile is able to distinguish the individual isomerization events. As can be seen in Fig. 2b (region II), the step-by-step progress of the single-molecule reaction – including the first isomerization that leads to a *cis*–*trans* partial product, and the second isomerization that results in the *trans*–*trans* final product – can be monitored by tracking changes in the electron transmission through the junction. The diradical intermediate that is sketched in Fig. 1a, which allows the molecular rearrangement after the force-induced ring opening of each individual cyclopropane fragment, is short-lived and not visible in the conductance or force profile. In addition to the isomerization events, a nonreactive conformational change can be seen in Fig. 2 at 26.6 Å as a drop in the conductance. Such a change leads to *cis*–*trans* intermediates with high and low conductance (*cis*–*trans* 1 and 2, respectively).

**Fig. 3:**
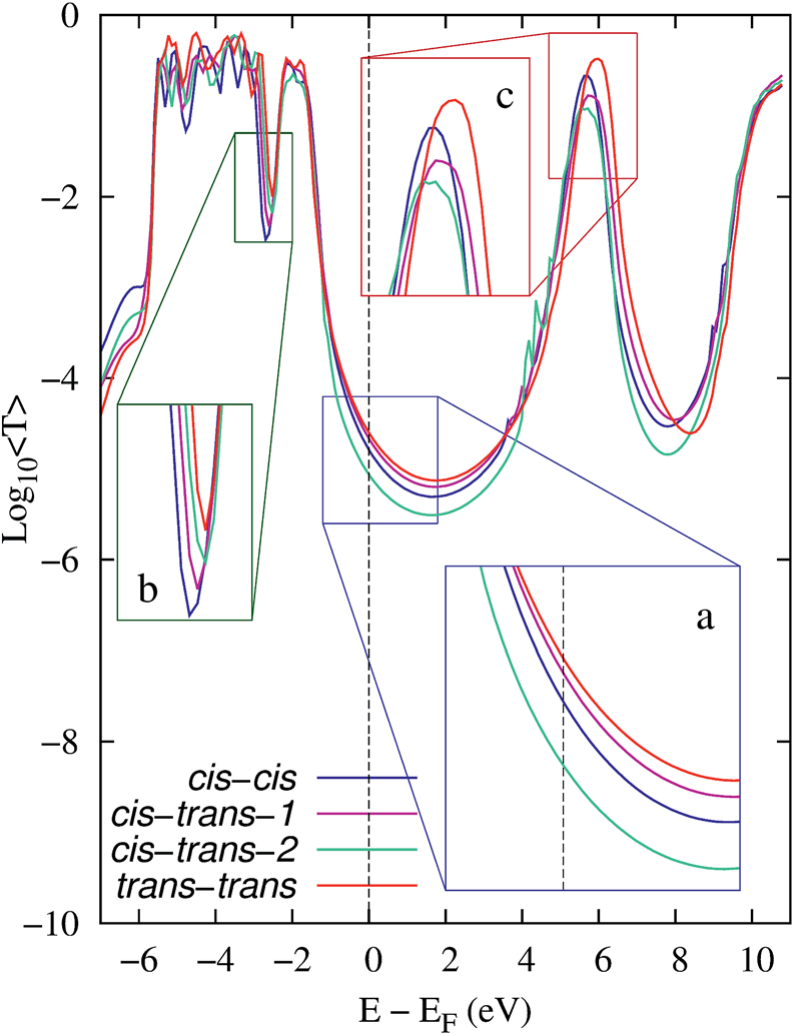
Spectra of the average EH transmission of the reactants (*cis*–*cis*), intermediates with high and low conductance (*cis*–*trans* 1 and 2) and products (*trans*–*trans*) of the 3→4 reaction. The *armchair* GNR was used as an electrode. 2000 molecular snapshots were considered when computing averages for the reactants, products and intermediate-1, and 790 snapshots for the intermediate-2. The insets detail three regions of the spectra that show (a) the average differences of transmission around the Fermi energy of the electrodes and the average shiftings of (b) the internal energy levels and (c) LUMO's energy.

Fig. 3 shows the average EH transmission spectra of the reactants (*cis*–*cis*), intermediates with high and low conductance (*cis*–*trans* 1 and 2) and products (*trans*–*trans*) of the 3→4 reaction. As detailed in the inset (a), the average transmission around the electrodes' Fermi energy distinguishes all species involved in the 3→4 reaction. This allowed us to monitor the step-by-step reaction progress using conductance. Fig. S1 and S2 (ESI†) show the average orbital energy during elongation. The HOMO and HOMO–1 orbitals are located at *E* – *E*_F_ ≈ –0.8→–0.6 eV and have a low transmission as they are partially localized in one of the extremes of the molecule. The transmission peak at *E* – *E*_F_ ≈ –2 eV is due to the HOMO–2 to HOMO–5 orbitals, and the peak at ∼–3 eV is due to HOMO–6. In turn, the contributions to transport by the LUMO and LUMO+1 orbitals are at *E* – *E*_F_ ≈ 5.8 eV.

The observed transmission changes at *E*_F_ during the reaction are the result of several conflicting contributing factors that include (i) an increase in the broadening of the transmission peak due to the HOMO–6 (Fig. 3b) and that due to the HOMO/HOMO–1 orbitals (the latter one is not evident in the averages in Fig. 3) which enhances *T*(*E*_F_), and (ii) a decrease in the HOMO–*n* orbital energies for *n* = 0→4 (see Fig. S1†) which reduces *T*(*E*_F_). While the conformational dynamics leads to clear changes in the LUMO and LUMO+1 orbital energies (Fig. 3c, and S2†), these changes are not expected to be determinants for *T*(*E*_F_) as transport in this case is governed by the HOMO levels.

**Fig. 4:**
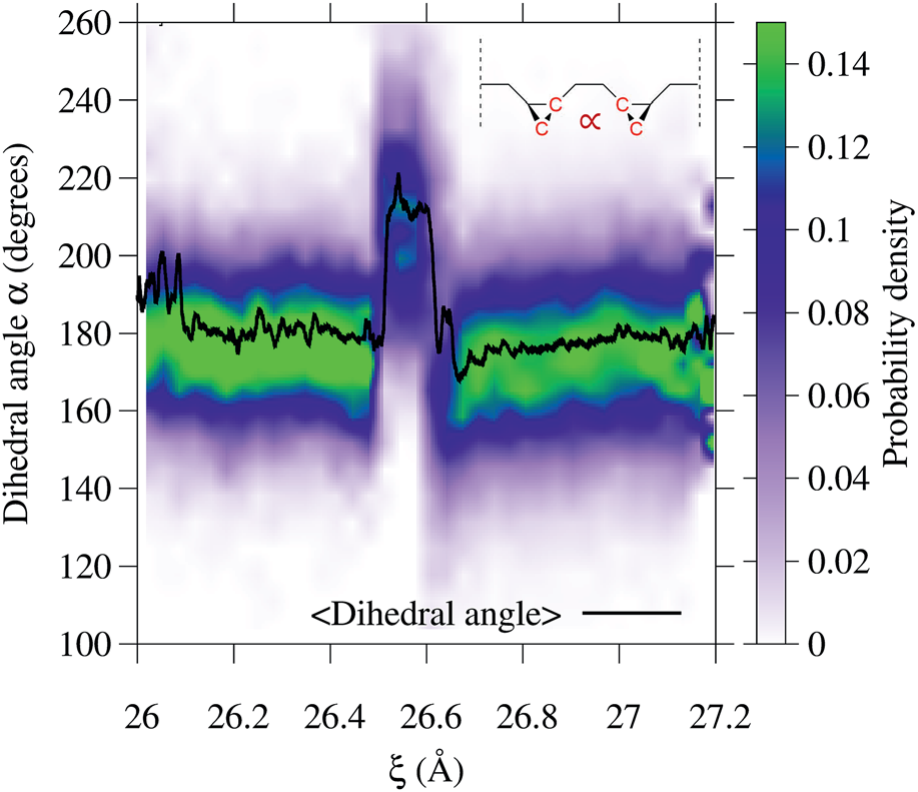
Variation of the dihedral angle *α* between the two rings during the 3→4 reaction. The dihedral angle is that defined by the four carbon atoms highlighted in the inset. Both isomerization events can be seen as a change in the average value of the dihedral angle between the two rings during the 3→4 reaction. The dihedral angle is that defined by the four carbon atoms highlighted in the inset. Both isomerization events can be seen as a change in the average value of the dihedral angle 〈*α*〉 (solid black line) and in the probability distribution (color code) at 26.5 and 26.7 Å. In addition, a nonreactive conformational change occurs at 26.6 Å. (solid black line) and in the probability distribution (color code) at 26.5 and 26.7 Å. In addition, a nonreactive conformational change occurs at 26.6 Å.

To gain additional insight into the conformational dynamics during the isomerization, we monitored the dihedral angle *α* between the two cyclopropane rings (Fig. 4). In addition to the isomerization events, the relative orientation of the rings changes during the dynamics. During event [circle containing 1] the rings go from an antiparallel configuration (*α* ≈ 180°) to a conformation with *α* ≈ 215°. The decay in the conductance at *ξ* ∼ 26.6 Å is associated with a non-reactive conformational change in which *α* goes from 215° to the most probable value of ∼165°. During event [circle containing 2], the most probable *α* goes back to the antiparallel configuration.

**Fig. 5:**
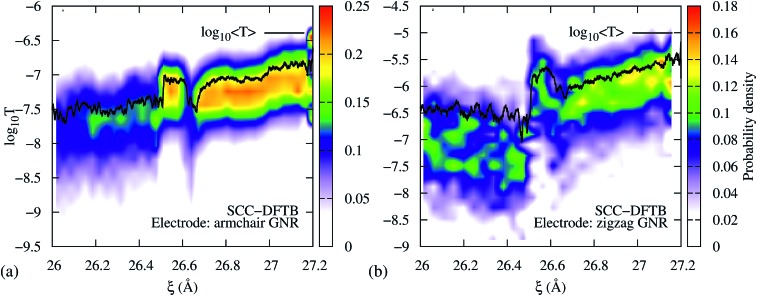
DFTB transmission during the 3→4 isomerization using semi-infinite (a) *armchair* and (b) *zigzag* GNR electrodes. In both cases, the isomerization steps are distinguishable in *T*(*E*_F_) *vs. ξ*. Further, the conductance profiles qualitatively agree with the extended Hückel results shown in Fig. 2.

To demonstrate that the results are robust to the choice of Hamiltonian model, we performed computations of the transmission during the 3→4 reaction using SCC-DFTB. Fig. 5a shows the resulting transmission profile obtained using an *armchair* GNR electrode, equivalent to the one that was used in the EH based method computations, and considering 0 K Fermi distributions for the electrodes (Fig. S3 in the ESI† shows that the results are equivalent to those for 300 K Fermi distributions). Although the DFTB *T*(*E*_F_) is 2.5 orders of magnitude lower than the EH values, the qualitative features during mechanical elongation are essentially identical. Therefore, we conclude that the trends are robust to the choice of Hamiltonian model.

To demonstrate that the results are robust to the choice of electrodes we performed computations of the transmission during the 3→4 reaction using metallic *zigzag* GNR electrodes (Fig. 5b) – as opposed to the semiconducting *armchair* electrode shown in Fig. 5a and 2b – gold electrodes (Fig. S4a, ESI†) and a GNR connected to the gold electrodes (Fig. S4b†). As can be seen, the intermediate steps in the isomerization reaction are clearly visible in all cases, indicating that the qualitative features of the *T*(*E*_F_) *vs. ξ* profile during the mechanically activated reaction are impervious to the choice of electrodes at low biases.

**Fig. 6:**
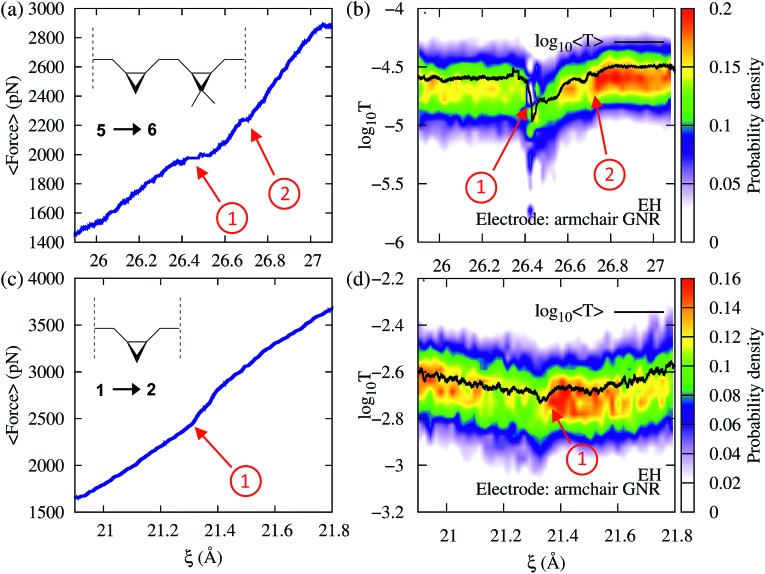
Force and EH transmission *vs.* extended-molecular length *ξ* during the *cis*-to-*trans* isomerization of (a and b) the system with two nonequivalent cycles 5→6 and (c and d) a single substituted cyclopropane 1→2. In (a) and (b) the first isomerization corresponds to the cycle with methyl substituents. In all cases a semi-infinite *armchair* GNR was used as an electrode. Note that the two events in the 5→6 isomerization are distinguishable in both transmission and force profiles. In turn, the 1→2 isomerization exhibits less pronounced changes in force and conductance.

To demonstrate that force provides useful complementary information to conductance, we now consider cases where the conductance changes are not pronounced during the mechanically induced isomerization. Consider first the force–transport profile during the 5→6 isomerization of a molecule with two non-equivalent cyclopropane-like rings shown in Fig. 6a and b. In this system, one of the rings is functionalized with two methyl groups, breaking the degeneracy in the molecule. Because the two rings require different forces to be mechanically open, both isomerizations can be seen as distinct events in the force–extension isotherm (Fig. 6a). The methyl substituted ring isomerizes at forces ∼250 pN below those required to isomerize the unsubstituted ring. By contrast, while the conductance clearly signals the first isomerization, the second event is not clearly visible. As in 3→4, the conductance profile in this case is the result of several contributing factors.

As an additional example, consider now the 1→2 isomerization of a single ring system as monitored using conductance and force (Fig. 6c and d). While the process produces very little change in conductance because the transmission of reactants and products is similar, it can be clearly identified in the force profile as a change in the elasticity of the junction. These results demonstrate that the correlation between force and conductance offers a highly discriminating window into the chemical changes during elongation, which go beyond what can be discerned through force or conductance alone.

## 4 Conclusions

In this contribution we have computationally demonstrated that the step-by-step progress of the mechanically activated *cis*-to-*trans* single-molecule isomerization of cyclopropane oligomers can be resolved using simultaneous measurements of force and conductance. The conductance profile can distinguish between isomerization steps that are not visible in the force profile, such as those that occur at the same force in the 3→4 reaction. By contrast, the force signals molecular deformations, before and after isomerization, which are not necessarily visible in the conductance profile, and isomerization events that are non-degenerate. The results illustrate the use of force–conductance measurements as a highly discriminating spectroscopy for monitoring chemical reactions at the single-molecule limit.

The conceptual advances of this paper show that (i) chemical reactions, including intermediates, can be monitored using conductance and force by merging covalent mechanochemistry with molecular conductance. (ii) The correlations between force and conductance are key to distinguishing events that occur at the same force or events that do not lead to appreciable changes in the conductance. In fact, such a correlation enabled us to distinguish reactants, products and intermediates even in an isomerization reaction that involves relatively subtle structural changes. These advances go beyond previous efforts to examine chemical reactions using force (see *e.g.*ref. 28 and 29) or conductance (see *e.g.*ref. 2, 32 and 47) alone and complement previous experimental^32^,^62^–^66^ and theoretical^42^–^46^,^67^–^72^ efforts to characterize the conductance properties of molecular junctions as they are mechanically manipulated.

The experimental implementation of such a mechanically induced transition can be realized in standard setups for molecular electronics experiments^56^ as it just requires the junction to be mechanically elongated. Determining the force, in addition to conductance, to monitor the transition in room temperature measurements can be realized using, for example, the conductive probe atomic force microscope (CP-AFM) setup.^73^ The qualitative features of the computational observations are within the observable conductance range,^74^ and were shown to be robust to the choice of electrode, electrode temperature, and Hamiltonian model. The main experimental challenge is thus to use electrodes and molecule–electrode anchor groups that can endure the ∼2 nN forces required to significantly reduce or even eliminate the activation barrier^15^–^17^,^20^ as needed to mechanically induce such reactions. Here, we employed GNR electrodes because they can endure such forces and because the chemistry to functionalize them as needed to anchor the main molecular backbone is relatively well developed.^75^

The combination of force and conductance measurements has the potential to develop into a powerful multidimensional single-molecule spectroscopy, and here these prospects were expanded into the realm of chemical reactivity.

## Conflicts of interest

There are no conflicts to declare.

## Supplementary Material

Supplementary information

## References

[cit1] Pavliček N., Gross L. (2017). Nat. Rev. Chem..

[cit2] Guan J., Jia C., Li Y., Liu Z., Wang J., Yang Z., Gu C., Su D., Houk K. N., Zhang D., Guo X. (2018). Sci. Adv..

[cit3] Weckbecker D., Coto P. B., Thoss M. (2017). Nano Lett..

[cit4] Schuler B., Fatayer S., Mohn F., Moll N., Pavliček N., Meyer G., Peña D., Gross L. (2016). Nat. Chem..

[cit5] Kumagai T., Hanke F., Gawinkowski S., Sharp J., Kotsis K., Waluk J., Persson M., Grill L. (2014). Nat. Chem..

[cit6] Zhao P., Wang P., Zhang Z., Liu D. (2010). Phys. B.

[cit7] Voigt N. V., Tørring T., Rotaru A., Jacobsen M. F., Ravnsbæk J. B., Subramani R., Mamdouh W., Kjems J., Mokhir A., Besenbacher F., Vestarager Gothelf K. (2010). Nat. Nanotechnol..

[cit8] Grill L., Dyer M., Lafferentz L., Persson M., Peters M. V., Hecht S. (2007). Nat. Nanotechnol..

[cit9] Del Valle M., Gutiérrez R., Tejedor C., Cuniberti G. (2007). Nat. Nanotechnol..

[cit10] Xu B. Q., Li X. L., Xiao X. Y., Sakaguchi H., Tao N. J. (2005). Nano Lett..

[cit11] Ho W. (2002). J. Chem. Phys..

[cit12] Kim Y., Komeda T., Kawai M. (2002). Phys. Rev. Lett..

[cit13] Hla S.-W., Bartels L., Meyer G., Rieder K.-H. (2000). Phys. Rev. Lett..

[cit14] Krupička M., Sander W., Marx D. (2014). J. Phys. Chem. Lett..

[cit15] Ribas-Arino J., Shiga M., Marx D. (2009). Angew. Chem..

[cit16] Ribas-Arino J., Shiga M., Marx D. (2009). Chem.–Eur. J..

[cit17] Dopieralski P., Ribas-Arino J., Marx D. (2011). Angew. Chem..

[cit18] Wang J., Kouznetsova T. B., Craig S. L. (2015). J. Am. Chem. Soc..

[cit19] Wang J., Kouznetsova T. B., Niu Z., Rheingold A. L., Craig S. L. (2015). J. Org. Chem..

[cit20] Wollenhaupt M., Krupička M., Marx D. (2015). ChemPhysChem.

[cit21] Gossweiler G. R., Kouznetsova T. B., Craig S. L. (2015). J. Am. Chem. Soc..

[cit22] Wang J., Kouznetsova T. B., Boulatov R., Craig S. L. (2016). Nat. Commun..

[cit23] Pill M. F., Holz K., Preußke N., Berger F., Clausen-Schaumann H., Lüning U., Beyer M. K. (2016). Chem.–Eur. J..

[cit24] Konôpka M., Turanský R., Reichert J., Fuchs H., Marx D., Stich I. (2008). Phys. Rev. Lett..

[cit25] Ribas-Arino J., Marx D. (2012). Chem. Rev..

[cit26] Akbulatov S., Boulatov R. (2017). ChemPhysChem.

[cit27] Lenhardt J. M., Ong M. T., Choe R., Evenhuis C. R., Martinez T. J., Craig S. L. (2010). Science.

[cit28] Wang J., Kouznetsova T. B., Niu Z., Ong M. T., Klukovich H. M., Rheingold A. L., Martinez T. J., Craig S. L. (2015). Nat. Chem..

[cit29] Wang J., Kouznetsova T. B., Craig S. L. (2016). J. Am. Chem. Soc..

[cit30] Li H., Walker G. C. (2017). ACS Nano.

[cit31] Leary E., Roche C., Jiang H.-W., Grace I., GonzaÌlez M. T., Rubio-Bollinger G., Romero-MunÌfiz C., Xiong Y., Al-Galiby Q., Noori M., Lebedeva M. A., Porfyrakis K., Agrait N., Hodgson A., Higgins S. J., Lambert C. J., Anderson H. L., Nichols R. J. (2018). J. Am. Chem. Soc..

[cit32] Li Y., Haworth N. L., Xiang L., Ciampi S., Coote M. L., Tao N. (2017). J. Am. Chem. Soc..

[cit33] Koti Ainavarapu S. R., Wiita A. P., Dougan L., Uggerud E., Fernandez J. M. (2008). J. Am. Chem. Soc..

[cit34] Konôpka M., Turanský R., Dubecký M., Marx D., Stich I. (2009). J. Phys. Chem. C.

[cit35] Turanský R., Konôpka M., Doltsinis N. L., Štich I., Marx D. (2010). ChemPhysChem.

[cit36] Konda S. S. M., Brantley J. N., Bielawski C. W., Makarov D. E. (2011). J. Chem. Phys..

[cit37] Dopieralski P., Ribas-Arino J., Anjukandi P., Krupička M., Kiss J., Marx D. (2013). Nat. Chem..

[cit38] Dopieralski P., Ribas-Arino J., Anjukandi P., Krupička M., Marx D. (2016). Angew. Chem..

[cit39] Dopieralski P., Ribas-Arino J., Anjukandi P., Krupička M., Marx D. (2017). Nat. Chem..

[cit40] Quapp W., Bofill J. M. (2016). J. Comput. Chem..

[cit41] Quapp W., Bofill J. M., Ribas-Ariño J. (2017). J. Phys. Chem. A.

[cit42] Franco I., Schatz G. C., Ratner M. A. (2009). J. Chem. Phys..

[cit43] Franco I., Schatz G. C. and Ratner M. A., in Nano and Cell Mechanics: Fundamentals and Frontiers, ed. H. D. Espinosa and G. Bao, John Wiley & Sons, 2012, ch. 14, pp. 359–388.

[cit44] Mejía L., Renaud N., Franco I. (2018). J. Phys. Chem. Lett..

[cit45] Li Z., Tkatchenko A., Franco I. (2018). J. Phys. Chem. Lett..

[cit46] Pirrotta A., De Vico L., Solomon G. C., Franco I. (2017). J. Chem. Phys..

[cit47] Jia C., Migliore A., Xin N., Huang S., Wang J., Yang Q., Wang S., Chen H., Wang D., Feng B., Liu Z., Zhang G., Qu D.-H., Tian H., Ratner M. A., Xu H., Nitzan A., Guo X. (2016). Science.

[cit48] Frank I., Tanenbaum D. M., van der Zande A. M., McEuen P. L. (2007). J. Vac. Sci. Technol., B: Microelectron. Nanometer Struct.--Process., Meas., Phenom..

[cit49] Faccio R., Denis P. A., Pardo H., Goyenola C., Mombrú A. W. (2009). J. Phys.: Condens. Matter.

[cit50] Plimpton S. (1995). J. Comput. Phys..

[cit51] Schneider T., Stoll E. (1978). Phys. Rev. B.

[cit52] Strachan A., van Duin A. C., Chakraborty D., Dasgupta S., Goddard III W. A. (2003). Phys. Rev. Lett..

[cit53] Van Duin A. C., Dasgupta S., Lorant F., Goddard W. A. (2001). J. Phys. Chem. A.

[cit54] Earl D. J., Deem M. W. (2005). Phys. Chem. Chem. Phys..

[cit55] Datta S., Quantum Transport: Atom to Transistor, Cambridge University Press, 2005.

[cit56] Cuevas J. C. and Scheer E., Molecular Electronics: an Introduction to Theory and Experiment, World Scientific, 2010, vol. 1.

[cit57] Hoffmann R. (1963). J. Chem. Phys..

[cit58] Hutcheson J., Franco I., Renaud N., Carignano M., Ratner M. A. and Schatz G. C., TRANSpull: Computes Pulling Coupled to Transport Properties of Single Molecules, 2011.

[cit59] Elstner M., Porezag D., Jungnickel G., Elsner J., Haugk M., Frauenheim T., Suhai S., Seifert G. (1998). Phys. Rev. B.

[cit60] Verzijl C., Seldenthuis J., Thijssen J. (2013). J. Chem. Phys..

[cit61] Aradi B., Hourahine B., Frauenheim T. (2007). J. Phys. Chem. A.

[cit62] Su T. A., Li H., Steigerwald M. L., Venkataraman L., Nuckolls C. (2015). Nat. Chem..

[cit63] Quek S. Y., Kamenetska M., Steigerwald M. L., Choi H. J., Louie S. G., Hybertsen M. S., Neaton J., Venkataraman L. (2009). Nat. Nanotechnol..

[cit64] Frei M., Aradhya S. V., Koentopp M., Hybertsen M. S., Venkataraman L. (2011). Nano Lett..

[cit65] Bruot C., Hihath J., Tao N. (2012). Nat. Nanotechnol..

[cit66] Rascón-Ramos H., Artés J. M., Li Y., Hihath J. (2015). Nat. Mater..

[cit67] Koch M., Li Z., Nacci C., Kumagai T., Franco I., Grill L. (2018). Phys. Rev. Lett..

[cit68] Carey R., Chen L., Gu B., Franco I. (2017). J. Chem. Phys..

[cit69] Pirrotta A., Solomon G. C., Franco I. (2016). J. Phys. Chem. C.

[cit70] Parker S. M., Smeu M., Franco I., Ratner M. A., Seideman T. (2014). Nano Lett..

[cit71] Franco I., Solomon G. C., Schatz G. C., Ratner M. A. (2011). J. Am. Chem. Soc..

[cit72] Franco I., George C. B., Solomon G. C., Schatz G. C., Ratner M. A. (2011). J. Am. Chem. Soc..

[cit73] Wold D. J., Frisbie C. D. (2001). J. Am. Chem. Soc..

[cit74] Lafferentz L., Ample F., Yu H., Hecht S., Joachim C., Grill L. (2009). Science.

[cit75] Georgakilas V., Otyepka M., Bourlinos A. B., Chandra V., Kim N., Kemp K. C., Hobza P., Zboril R., Kim K. S. (2012). Chem. Rev..

