# Supplementary material for: Force–conductance spectroscopy of a single-molecule reaction
Source: Chem Sci. 2019 Jan 25;10(11):3249–56. doi: 10.1039/c8sc04830d (PMC6429593; doi:10.1039/c8sc04830d)
Supplement: Supplementary file 1 [file SC-010-C8SC04830D-s001.pdf]

Electronic Supplementary Material (ESI) for Chemical Science.
This journal is © The Royal Society of Chemistry 2019

Supporting Information
Force-Conductance Spectroscopy of a Single-Molecule Reaction

Leopoldo Mejía[†] and Ignacio Franco[*,†,‡]

†*Department of Chemistry, University of Rochester, Rochester, New York 14627-0216, USA*

‡*Department of Physics, University of Rochester, Rochester, New York 14627-0216, USA*

E-mail: ignacio.franco@rochester.edu

Contents

1 Supporting figures **2**

2 Initial coordinates: 3 $\longrightarrow$ 4 reaction **4**

3 Initial coordinates: 1 $\longrightarrow$ 2 reaction **9**

4 Initial coordinates: 5 $\longrightarrow$ 6 reaction **14**

5 Example input for the mechanical pulling simulation in LAMMPS (3 $\longrightarrow$ 4 reaction) **19**

6 Example input for the electron transport computations using DFTB+ **25**

1 Supporting figures

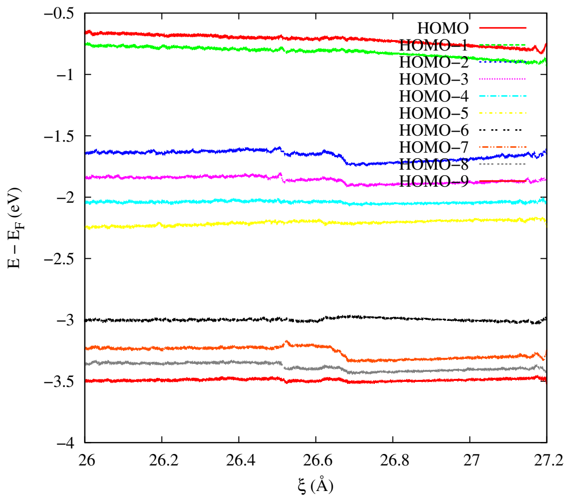

Figure S1: Variation of the average orbital energy for ten occupied frontier molecular orbitals during the **3** ⟶ **4** reaction. The computations were performed using the EH Hamiltonian.

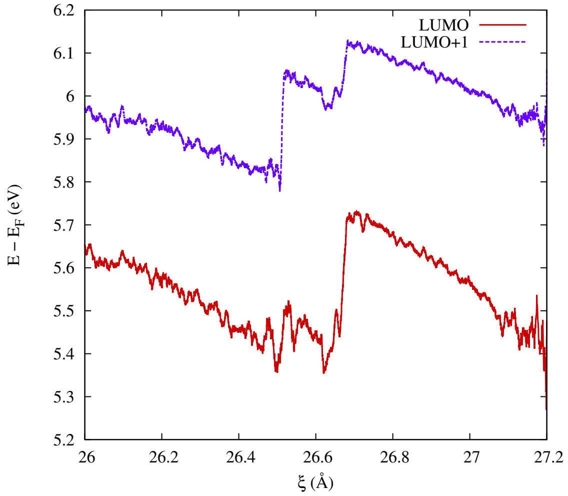

Figure S2: Variation of the average orbital energy for two unoccupied frontier molecular orbitals during the **3** ⟶ **4** reaction. The computations were performed using the EH Hamiltonian.

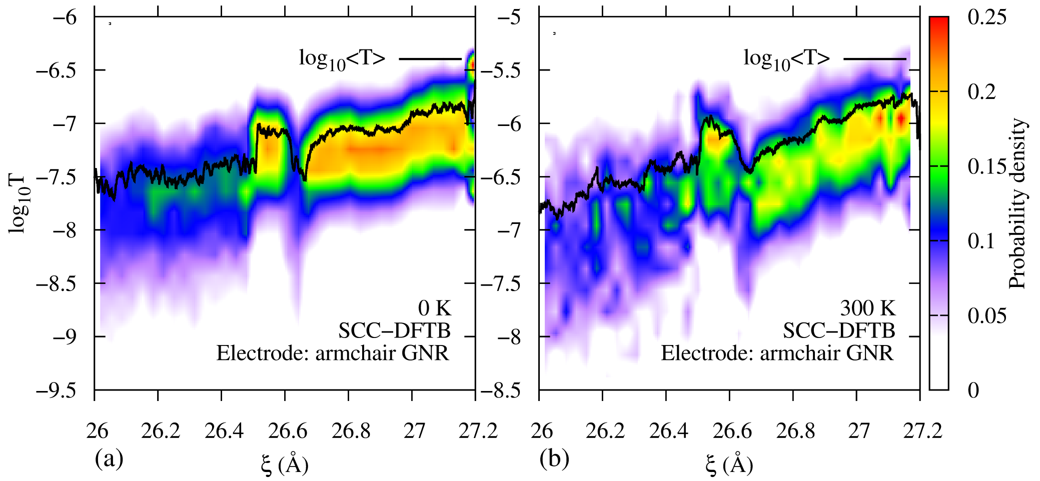

Figure S3: DFTB transmission during the mechanically-activated *cis*-to-*trans* isomerization of the system with two equivalent cycles **3** $\longrightarrow$ **4**, considering (a) 0 K and (b) 300 K Fermi distributions. In both cases a semi infinite *armchair* GNR was used as an electrode. The qualitative features of the conductance profiles are equivalent in both cases.

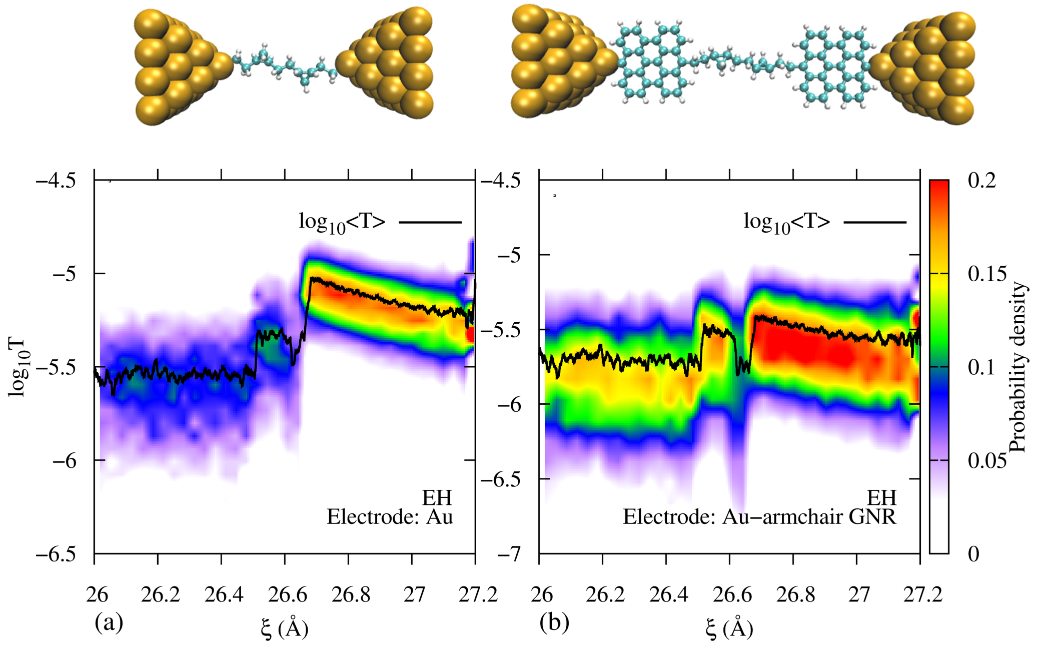

Figure S4: EH transmission during the mechanically-activated *cis*-to-*trans* isomerization of the system with two equivalent cycles **3** $\longrightarrow$ **4**, considering (a) gold and (b) GNR connected to gold electrodes. The reaction steps are resolved by the conductance regardless of the choice of electrodes.

2 Initial coordinates: 3 $\longrightarrow$ 4 reaction

LAMMPS data file. CGCMM style. atom_style full generated by VMD/TopoTools v1.5

114 atoms

0 bonds

0 angles

0 dihedrals

0 impropers

2 atom types

0 bond types

0 angle types

0 dihedral types

0 improper types

-24.000000 24.000000 xlo xhi

-21.539315 23.460685 ylo yhi

-27.932410 17.067590 zlo zhi

Pair Coeffs

#

1 CT

2 HC

Masses

1 12.011000 # CT

2 1.008000 # HC

Atoms

1 1 1 0.070000 -0.000000 -0.000000 0.000000 # CT

2 1 1 0.070000 -0.000000 0.000000 -10.864820 # CT

3 1 1 0.070000 -1.061440 0.891660 -3.800530 # CT

4 1 1 0.070000 -1.019610 0.751100 -2.255740 # CT

5 1 1 0.070000 -1.335010 2.073760 -2.901470 # CT

6 1 1 0.070000 0.220320 0.651200 -1.392190 # CT

7 1 2 0.060000 -1.851870 0.203880 -1.821150 # HC

8 1 2 0.060000 -0.591960 2.864210 -2.849200 # HC

9 1 2 0.060000 -2.352120 2.443520 -2.835770 # HC

10 1 2 0.060000 -1.927270 0.454210 -4.289330 # HC

11 1 1 0.070000 0.125500 0.982340 -4.740770 # CT

12 1 1 0.070000 -0.905760 0.902970 -8.593270 # CT

13 1 1 0.070000 -1.034140 0.907930 -7.053660 # CT

14 1 1 0.070000 -0.737260 2.178850 -7.795600 # CT

15 1 2 0.060000 0.254860 2.607600 -7.697660 # HC

16 1 2 0.060000 -1.519200 2.924510 -7.888840 # HC

17 1 2 0.060000 -0.490600 -0.972340 -0.126570 # HC

18 1 2 0.060000 1.069160 -0.237500 0.011470 # HC

19 1 2 0.060000 0.670980 1.640820 -1.252540 # HC

20 1 2 0.060000 0.953750 0.034860 -1.924390 # HC

21 1 1 0.070000 -0.044600 0.267990 -6.109660 # CT

22 1 2 0.060000 -2.045650 0.803980 -6.668000 # HC

23 1 2 0.060000 0.939400 0.224190 -6.589830 # HC

24 1 2 0.060000 -0.361590 -0.767930 -5.937920 # HC

25 1 2 0.060000 0.995580 0.539450 -4.243920 # HC

26 1 2 0.060000 0.364520 2.035830 -4.928760 # HC

27 1 1 0.070000 0.285280 0.474840 -9.420080 # CT

28 1 2 0.060000 1.245300 -0.114750 -10.931990 # HC

29 1 2 0.060000 -0.364930 -1.003140 -10.632030 # HC

30 1 2 0.060000 1.013220 1.294090 -9.469940 # HC

31 1 2 0.060000 0.791680 -0.352030 -8.905430 # HC

32 1 2 0.060000 -1.842880 0.742230 -9.113790 # HC

33 1 1 0.070000 -3.707310 0.021990 3.437190 # CT

34 1 1 0.070000 -3.705900 0.026480 2.010490 # CT

35 1 1 0.070000 -2.471010 0.018750 1.298170 # CT

36 1 1 0.070000 -2.473030 0.009770 4.150230 # CT

37 1 1 0.070000 -1.236340 -0.001700 3.437440 # CT

38 1 1 0.070000 -1.235320 0.003830 2.012060 # CT

39 1 1 0.070000 0.000000 -0.000000 1.300000 # CT

40 1 1 0.070000 -0.000640 -0.012400 4.149310 # CT

41 1 1 0.070000 1.235090 -0.019560 3.438420 # CT

42 1 1 0.070000 1.234620 -0.012880 2.013180 # CT

43 1 1 0.070000 2.470650 -0.013600 1.300510 # CT

44 1 1 0.070000 2.470480 -0.028710 4.152800 # CT

45 1 1 0.070000 3.705070 -0.039240 3.440590 # CT

46 1 1 0.070000 3.704830 -0.029790 2.013910 # CT

47 1 2 0.060000 2.471260 -0.002400 0.268570 # HC

48 1 2 0.060000 4.598630 -0.034680 1.498040 # HC

49 1 2 0.060000 4.598670 -0.053700 3.956620 # HC

50 1 2 0.060000 -4.601340 0.027590 3.952650 # HC

51 1 2 0.060000 -4.599240 0.035320 1.493880 # HC

52 1 2 0.060000 -2.484300 0.043320 0.202640 # HC

53 1 1 0.070000 3.707310 0.021990 -14.302010 # CT

54 1 1 0.070000 3.705900 0.026480 -12.875310 # CT

55 1 1 0.070000 2.471010 0.018750 -12.162990 # CT

56 1 1 0.070000 2.473030 0.009770 -15.015050 # CT

57 1 1 0.070000 1.236340 -0.001700 -14.302260 # CT

58 1 1 0.070000 1.235320 0.003830 -12.876880 # CT

59 1 1 0.070000 -0.000000 -0.000000 -12.164820 # CT

60 1 1 0.070000 0.000640 -0.012400 -15.014130 # CT

61 1 1 0.070000 -1.235090 -0.019560 -14.303240 # CT

62 1 1 0.070000 -1.234620 -0.012880 -12.878000 # CT

63 1 1 0.070000 -2.470650 -0.013600 -12.165330 # CT

64 1 1 0.070000 -2.470480 -0.028710 -15.017620 # CT

65 1 1 0.070000 -3.705070 -0.039240 -14.305410 # CT

66 1 1 0.070000 -3.704830 -0.029790 -12.878730 # CT

67 1 2 0.060000 -2.471260 -0.002400 -11.133390 # HC

68 1 2 0.060000 -4.598630 -0.034680 -12.362860 # HC

69 1 2 0.060000 -4.598670 -0.053700 -14.821440 # HC

70 1 2 0.060000 4.601340 0.027590 -14.817470 # HC

71 1 2 0.060000 4.599240 0.035320 -12.358700 # HC

72 1 2 0.060000 2.484300 0.043320 -11.067460 # HC

73 1 2 0.060000 -4.669070 0.039210 5.737610 # HC

74 1 2 0.060000 -4.670600 0.064510 8.269880 # HC

75 1 2 0.060000 -2.481550 0.074680 9.542520 # HC

76 1 2 0.060000 -0.000390 0.042570 9.542080 # HC

77 1 2 0.060000 4.665030 -0.050840 5.740470 # HC

78 1 2 0.060000 4.663310 -0.058450 8.273450 # HC

79 1 2 0.060000 2.471990 -0.018410 9.541710 # HC

80 1 2 0.060000 -4.665030 -0.050840 -16.605289 # HC

81 1 2 0.060000 -2.471990 -0.018410 -20.406530 # HC

82 1 2 0.060000 -4.663310 -0.058450 -19.138269 # HC

83 1 2 0.060000 0.000390 0.042570 -20.406900 # HC

84 1 2 0.060000 4.669070 0.039210 -16.602430 # HC

85 1 2 0.060000 4.670600 0.064510 -19.134701 # HC

86 1 2 0.060000 2.481550 0.074680 -20.407339 # HC

87 1 1 0.070000 -3.709770 0.055950 7.718160 # CT

88 1 1 0.070000 -3.708970 0.037990 6.290670 # CT

89 1 1 0.070000 -2.476740 0.018510 5.575060 # CT

90 1 1 0.070000 -2.480410 0.062900 8.434580 # CT

91 1 1 0.070000 -1.244190 0.054600 7.717610 # CT

92 1 1 0.070000 -1.240110 0.022240 6.287970 # CT

93 1 1 0.070000 -0.000000 0.000000 5.576300 # CT

94 1 1 0.070000 -0.000990 0.039560 8.434080 # CT

95 1 1 0.070000 1.240550 0.020610 7.716860 # CT

96 1 1 0.070000 1.237470 0.005270 6.290620 # CT

97 1 1 0.070000 2.472400 -0.022750 5.577810 # CT

98 1 1 0.070000 2.472850 -0.012650 8.433730 # CT

99 1 1 0.070000 3.703660 -0.038410 7.719970 # CT

100 1 1 0.070000 3.704730 -0.038510 6.293040 # CT

101 1 1 0.070000 3.709770 0.055950 -18.582979 # CT

102 1 1 0.070000 3.708970 0.037990 -17.155491 # CT

103 1 1 0.070000 2.476740 0.018510 -16.439880 # CT

104 1 1 0.070000 2.480410 0.062900 -19.299400 # CT

105 1 1 0.070000 1.244190 0.054600 -18.582430 # CT

106 1 1 0.070000 1.240110 0.022240 -17.152790 # CT

107 1 1 0.070000 0.000000 0.000000 -16.441120 # CT

108 1 1 0.070000 0.000990 0.039560 -19.298901 # CT

109 1 1 0.070000 -1.240550 0.020610 -18.581680 # CT

110 1 1 0.070000 -1.237470 0.005270 -17.155439 # CT

111 1 1 0.070000 -2.472400 -0.022750 -16.442631 # CT

112 1 1 0.070000 -2.472850 -0.012650 -19.298550 # CT

113 1 1 0.070000 -3.703660 -0.038410 -18.584789 # CT

114 1 1 0.070000 -3.704730 -0.038510 -17.157860 # CT

3 Initial coordinates: 1 $\longrightarrow$ 2 reaction

LAMMPS data file. CGCMM style. atom_style full generated by VMD/TopoTools v1.5

101 atoms

2 atom types

-24.000000 24.000000 xlo xhi

-20.959225 24.040775 ylo yhi

-20.093030 24.906970 zlo zhi

Pair Coeffs

#

1 CT

2 HC

Masses

1 12.011000 # CT

2 1.008000 # HC

Atoms

1 1 1 0.000000 -0.180390 1.891270 3.091320 # CT

2 1 1 0.000000 0.693520 3.001620 2.475670 # CT

3 1 1 0.000000 -0.044410 2.039480 1.572920 # CT

4 1 2 0.000000 1.791850 2.820910 2.469090 # HC

5 1 2 0.000000 0.253310 3.965160 2.810980 # HC

6 1 1 0.000000 0.820140 1.018620 0.823060 # CT

7 1 2 0.000000 -0.748770 2.678650 1.012800 # HC

8 1 2 0.000000 -1.175630 2.259740 3.422030 # HC

9 1 1 0.000000 0.683450 1.229670 4.187920 # CT

10 1 1 0.000000 0.000000 0.000000 0.000000 # CT

11 1 2 0.000000 1.438490 0.455240 1.553160 # HC

12 1 2 0.000000 1.513790 1.550500 0.135550 # HC

13 1 1 0.000000 -0.000000 0.000000 4.813930 # CT

14 1 2 0.000000 0.780700 2.022740 4.959880 # HC

15 1 2 0.000000 1.715480 0.957770 3.871600 # HC

16 1 2 0.000000 -0.748820 -0.517130 0.637550 # HC

17 1 2 0.000000 0.734450 -0.770180 -0.321190 # HC

18 1 2 0.000000 0.195120 -0.883610 4.166610 # HC

19 1 2 0.000000 -1.100300 0.146380 4.861870 # HC

20 1 1 0.000000 -3.707310 0.021990 8.251120 # CT

21 1 1 0.000000 -3.705900 0.026480 6.824420 # CT

22 1 1 0.000000 -2.471010 0.018750 6.112110 # CT

23 1 1 0.000000 -2.473030 0.009770 8.964160 # CT

24 1 1 0.000000 -1.236340 -0.001700 8.251370 # CT

25 1 1 0.000000 -1.235320 0.003830 6.825990 # CT

26 1 1 0.000000 0.000000 -0.000000 6.113930 # CT

27 1 1 0.000000 -0.000640 -0.012400 8.963240 # CT

28 1 1 0.000000 1.235090 -0.019560 8.252350 # CT

29 1 1 0.000000 1.234620 -0.012880 6.827120 # CT

30 1 1 0.000000 2.470650 -0.013600 6.114440 # CT

31 1 1 0.000000 2.470480 -0.028710 8.966730 # CT

32 1 1 0.000000 3.705070 -0.039240 8.254520 # CT

33 1 1 0.000000 3.704830 -0.029790 6.827840 # CT

34 1 2 0.000000 2.471260 -0.002400 5.082500 # HC

35 1 2 0.000000 4.598630 -0.034680 6.311970 # HC

36 1 2 0.000000 4.598670 -0.053700 8.770550 # HC

37 1 2 0.000000 -4.601340 0.027590 8.766580 # HC

38 1 2 0.000000 -4.599240 0.035320 6.307810 # HC

39 1 2 0.000000 -2.484300 0.043320 5.016570 # HC

40 1 1 0.000000 3.707310 0.021990 -3.437190 # CT

41 1 1 0.000000 3.705900 0.026480 -2.010490 # CT

42 1 1 0.000000 2.471010 0.018750 -1.298170 # CT

43 1 1 0.000000 2.473030 0.009770 -4.150230 # CT

44 1 1 0.000000 1.236340 -0.001700 -3.437440 # CT

45 1 1 0.000000 1.235320 0.003830 -2.012060 # CT

46 1 1 0.000000 -0.000000 -0.000000 -1.300000 # CT

47 1 1 0.000000 0.000640 -0.012400 -4.149310 # CT

48 1 1 0.000000 -1.235090 -0.019560 -3.438420 # CT

49 1 1 0.000000 -1.234620 -0.012880 -2.013180 # CT

50 1 1 0.000000 -2.470650 -0.013600 -1.300510 # CT

51 1 1 0.000000 -2.470480 -0.028710 -4.152800 # CT

52 1 1 0.000000 -3.705070 -0.039240 -3.440590 # CT

53 1 1 0.000000 -3.704830 -0.029790 -2.013910 # CT

54 1 2 0.000000 -2.471260 -0.002400 -0.268570 # HC

55 1 2 0.000000 -4.598630 -0.034680 -1.498040 # HC

56 1 2 0.000000 -4.598670 -0.053700 -3.956620 # HC

57 1 2 0.000000 4.601340 0.027590 -3.952650 # HC

58 1 2 0.000000 4.599240 0.035320 -1.493880 # HC

59 1 2 0.000000 2.484300 0.043320 -0.202640 # HC

60 1 2 0.000000 4.597480 -0.057060 13.049420 # HC

61 1 2 0.000000 2.472050 -0.018020 14.279650 # HC

62 1 2 0.000000 -0.000430 0.042380 14.280020 # HC

63 1 2 0.000000 4.599160 -0.050010 10.592310 # HC

64 1 2 0.000000 -4.603210 0.039120 10.589470 # HC

65 1 2 0.000000 -4.604700 0.063920 13.045970 # HC

66 1 2 0.000000 -2.481480 0.073880 14.280460 # HC

67 1 2 0.000000 -4.599160 -0.050010 -5.778380 # HC

68 1 2 0.000000 -4.597480 -0.057060 -8.235490 # HC

69 1 2 0.000000 -2.472050 -0.018020 -9.465720 # HC

70 1 2 0.000000 0.000430 0.042380 -9.466080 # HC

71 1 2 0.000000 2.481480 0.073880 -9.466520 # HC

72 1 2 0.000000 4.604700 0.063920 -8.232030 # HC

73 1 2 0.000000 4.603210 0.039120 -5.775540 # HC

74 1 1 0.000000 -3.709770 0.055950 12.532100 # CT

75 1 1 0.000000 -3.708970 0.037990 11.104600 # CT

76 1 1 0.000000 -2.476740 0.018510 10.388990 # CT

77 1 1 0.000000 -2.480410 0.062900 13.248520 # CT

78 1 1 0.000000 -1.244190 0.054600 12.531540 # CT

79 1 1 0.000000 -1.240110 0.022240 11.101900 # CT

80 1 1 0.000000 -0.000000 0.000000 10.390230 # CT

81 1 1 0.000000 -0.000990 0.039560 13.248020 # CT

82 1 1 0.000000 1.240550 0.020610 12.530790 # CT

83 1 1 0.000000 1.237470 0.005270 11.104550 # CT

84 1 1 0.000000 2.472400 -0.022750 10.391740 # CT

85 1 1 0.000000 2.472850 -0.012650 13.247660 # CT

86 1 1 0.000000 3.703660 -0.038410 12.533900 # CT

87 1 1 0.000000 3.704730 -0.038510 11.106970 # CT

88 1 1 0.000000 3.709770 0.055950 -7.718160 # CT

89 1 1 0.000000 3.708970 0.037990 -6.290670 # CT

90 1 1 0.000000 2.476740 0.018510 -5.575060 # CT

91 1 1 0.000000 2.480410 0.062900 -8.434580 # CT

92 1 1 0.000000 1.244190 0.054600 -7.717610 # CT

93 1 1 0.000000 1.240110 0.022240 -6.287970 # CT

94 1 1 0.000000 0.000000 0.000000 -5.576300 # CT

95 1 1 0.000000 0.000990 0.039560 -8.434080 # CT

96 1 1 0.000000 -1.240550 0.020610 -7.716860 # CT

97 1 1 0.000000 -1.237470 0.005270 -6.290620 # CT

98 1 1 0.000000 -2.472400 -0.022750 -5.577810 # CT

99 1 1 0.000000 -2.472850 -0.012650 -8.433730 # CT

100 1 1 0.000000 -3.703660 -0.038410 -7.719970 # CT

101 1 1 0.000000 -3.704730 -0.038510 -6.293040 # CT

4 Initial coordinates: 5 $\longrightarrow$ 6 reaction

LAMMPS data file. CGCMM style. atom_style full generated by VMD/TopoTools v1.7

120 atoms

0 bonds

0 angles

0 dihedrals

0 impropers

2 atom types

0 bond types

0 angle types

0 dihedral types

0 improper types

-24.000000 24.000000 xlo xhi

-23.053625 21.946375 ylo yhi

-17.118435 27.881565 zlo zhi

Pair Coeffs

#

1 CT

2 HC

Masses

1 12.011000 # CT

2 1.008000 # HC

Atoms

1 1 1 0.070000 0.000000 0.000000 0.000000 # CT

2 1 1 0.070000 0.000000 0.000000 10.763130 # CT

3 1 1 0.070000 0.001480 -1.200190 2.249390 # CT

4 1 1 0.070000 0.105870 -1.214690 3.764750 # CT

5 1 1 0.070000 -0.144110 0.002200 4.609080 # CT

6 1 1 0.070000 0.044480 -0.297960 6.105450 # CT

7 1 2 0.060000 -1.167070 0.359070 4.438870 # HC

8 1 2 0.060000 0.527360 0.817270 4.312430 # HC

9 1 1 0.070000 -0.089650 0.946820 6.932760 # CT

10 1 2 0.060000 1.041260 -0.725950 6.266720 # HC

11 1 2 0.060000 -0.680700 -1.058560 6.419610 # HC

12 1 1 0.070000 -0.069960 0.930740 8.450940 # CT

13 1 1 0.070000 0.051890 -0.325770 9.265740 # CT

14 1 2 0.060000 -0.917070 0.555640 10.993050 # HC

15 1 2 0.060000 0.838750 0.657320 11.023900 # HC

16 1 1 0.070000 -0.403790 0.021880 1.480450 # CT

17 1 2 0.060000 0.017090 0.929210 1.930240 # HC

18 1 2 0.060000 -1.493990 0.119970 1.548270 # HC

19 1 2 0.060000 1.054930 0.281280 -0.098550 # HC

20 1 2 0.060000 -0.581260 0.935750 -0.018670 # HC

21 1 1 0.070000 1.351190 -1.304550 2.910650 # CT

22 1 2 0.060000 -0.426030 -2.100360 1.820220 # HC

23 1 1 0.070000 2.247540 -0.166630 2.851100 # CT

24 1 1 0.070000 2.119300 -2.537050 2.957250 # CT

25 1 2 0.060000 -0.256590 -2.119360 4.241840 # HC

26 1 2 0.060000 1.006380 -0.813070 9.034400 # HC

27 1 2 0.060000 -0.744700 -1.035720 9.014130 # HC

28 1 1 0.070000 -1.352730 1.226720 7.710900 # CT

29 1 2 0.060000 0.419320 1.788460 6.473900 # HC

30 1 2 0.060000 0.456970 1.760830 8.910880 # HC

31 1 2 0.060000 -2.137940 0.479880 7.713540 # HC

32 1 2 0.060000 -1.721380 2.245760 7.732550 # HC

33 1 1 0.070000 -3.709770 0.055950 18.656290 # CT

34 1 1 0.070000 -3.708970 0.037990 17.228800 # CT

35 1 1 0.070000 -2.476740 0.018510 16.513189 # CT

36 1 1 0.070000 -2.480410 0.062900 19.372709 # CT

37 1 1 0.070000 -1.244190 0.054600 18.655741 # CT

38 1 1 0.070000 -1.240110 0.022240 17.226101 # CT

39 1 1 0.070000 -0.000000 0.000000 16.514429 # CT

40 1 1 0.070000 1.240550 0.020610 18.654989 # CT

41 1 1 0.070000 2.472400 -0.022750 16.515940 # CT

42 1 1 0.070000 -0.000990 0.039560 19.372210 # CT

43 1 1 0.070000 1.237470 0.005270 17.228750 # CT

44 1 1 0.070000 2.472850 -0.012650 19.371861 # CT

45 1 1 0.070000 3.703660 -0.038410 18.658100 # CT

46 1 1 0.070000 3.704730 -0.038510 17.231171 # CT

47 1 1 0.070000 -3.707310 0.021990 14.375320 # CT

48 1 1 0.070000 -0.000640 -0.012400 15.087440 # CT

49 1 1 0.070000 -3.705900 0.026480 12.948620 # CT

50 1 1 0.070000 -2.471010 0.018750 12.236300 # CT

51 1 1 0.070000 -2.473030 0.009770 15.088360 # CT

52 1 1 0.070000 -1.236340 -0.001700 14.375570 # CT

53 1 1 0.070000 -1.235320 0.003830 12.950190 # CT

54 1 1 0.070000 0.000000 -0.000000 12.238130 # CT

55 1 1 0.070000 1.235090 -0.019560 14.376550 # CT

56 1 1 0.070000 1.234620 -0.012880 12.951310 # CT

57 1 1 0.070000 2.470650 -0.013600 12.238640 # CT

58 1 1 0.070000 2.470480 -0.028710 15.090930 # CT

59 1 1 0.070000 3.705070 -0.039240 14.378720 # CT

60 1 1 0.070000 3.704830 -0.029790 12.952040 # CT

61 1 2 0.060000 2.471260 -0.002400 11.206700 # HC

62 1 2 0.060000 -2.484300 0.043320 11.140770 # HC

63 1 2 0.060000 4.598630 -0.034680 12.436170 # HC

64 1 2 0.060000 4.598670 -0.053700 14.894750 # HC

65 1 2 0.060000 4.599160 -0.050000 16.716511 # HC

66 1 2 0.060000 4.597490 -0.057070 19.173611 # HC

67 1 2 0.060000 2.472040 -0.018010 20.403839 # HC

68 1 2 0.060000 -2.481480 0.073870 20.404659 # HC

69 1 2 0.060000 -4.604700 0.063910 19.170170 # HC

70 1 2 0.060000 -4.601340 0.027590 14.890780 # HC

71 1 2 0.060000 -4.599240 0.035320 12.432010 # HC

72 1 2 0.060000 -4.629490 0.171360 16.679430 # HC

73 1 2 0.060000 -0.000430 0.042360 20.404209 # HC

74 1 1 0.070000 3.709770 0.055950 -7.893160 # CT

75 1 1 0.070000 3.708970 0.037990 -6.465670 # CT

76 1 1 0.070000 2.480410 0.062900 -8.609580 # CT

77 1 1 0.070000 1.244190 0.054600 -7.892610 # CT

78 1 1 0.070000 1.240110 0.022240 -6.462970 # CT

79 1 1 0.070000 0.000990 0.039560 -8.609080 # CT

80 1 1 0.070000 -1.240550 0.020610 -7.891860 # CT

81 1 1 0.070000 -1.237470 0.005270 -6.465620 # CT

82 1 1 0.070000 -2.472850 -0.012650 -8.608730 # CT

83 1 1 0.070000 -3.703660 -0.038410 -7.894970 # CT

84 1 1 0.070000 -3.704730 -0.038510 -6.468040 # CT

85 1 1 0.070000 2.476740 0.018510 -5.750060 # CT

86 1 1 0.070000 0.000000 0.000000 -5.751300 # CT

87 1 1 0.070000 -2.472400 -0.022750 -5.752810 # CT

88 1 1 0.070000 3.707310 0.021990 -3.612190 # CT

89 1 1 0.070000 3.705900 0.026480 -2.185490 # CT

90 1 1 0.070000 2.471010 0.018750 -1.473170 # CT

91 1 1 0.070000 2.473030 0.009770 -4.325230 # CT

92 1 1 0.070000 1.236340 -0.001700 -3.612440 # CT

93 1 1 0.070000 1.235320 0.003830 -2.187060 # CT

94 1 1 0.070000 -0.000000 -0.000000 -1.475000 # CT

95 1 1 0.070000 0.000640 -0.012400 -4.324310 # CT

96 1 1 0.070000 -1.235090 -0.019560 -3.613420 # CT

97 1 1 0.070000 -1.234620 -0.012880 -2.188180 # CT

98 1 1 0.070000 -2.470650 -0.013600 -1.475510 # CT

99 1 1 0.070000 -2.470480 -0.028710 -4.327800 # CT

100 1 1 0.070000 -3.705070 -0.039240 -3.615590 # CT

101 1 1 0.070000 -3.704830 -0.029790 -2.188910 # CT

102 1 2 0.060000 -2.471260 -0.002400 -0.443570 # HC

103 1 2 0.060000 2.484300 0.043320 -0.377640 # HC

104 1 2 0.060000 -4.598630 -0.034680 -1.673040 # HC

105 1 2 0.060000 -4.598670 -0.053700 -4.131620 # HC

106 1 2 0.060000 -4.599160 -0.050000 -5.953380 # HC

107 1 2 0.060000 -4.597490 -0.057070 -8.410480 # HC

108 1 2 0.060000 -2.472040 -0.018010 -9.640710 # HC

109 1 2 0.060000 2.481480 0.073870 -9.641530 # HC

110 1 2 0.060000 4.604700 0.063910 -8.407040 # HC

111 1 2 0.060000 4.601340 0.027590 -4.127650 # HC

112 1 2 0.060000 4.599240 0.035320 -1.668880 # HC

113 1 2 0.060000 4.629490 0.171360 -5.916300 # HC

114 1 2 0.060000 0.000430 0.042360 -9.641080 # HC

115 1 2 0.060000 2.965280 -2.454870 2.307270 # HC

116 1 2 0.060000 1.503940 -3.353010 2.640320 # HC

117 1 2 0.060000 2.454330 -2.710880 3.958470 # HC

118 1 2 0.060000 3.071610 -0.395000 2.207930 # HC

119 1 2 0.060000 2.611590 0.052420 3.833130 # HC

120 1 2 0.060000 1.720980 0.682540 2.468280 # HC

5 Example input for the mechanical pulling simulation in LAMMPS (3 $\longrightarrow$ 4 reaction)

#initial

units real

boundary s s s

atom_style full

#atom definition

read_data data.mol_pull

```
pair_style reax/c lmp_control
pair_coeff * * ffield.reax C H

compute reax all pair reax/c

variable eb equal c_reax[1]
variable ea equal c_reax[2]
variable elp equal c_reax[3]
variable emol equal c_reax[4]
variable ev equal c_reax[5]
variable epen equal c_reax[6]
variable ecoa equal c_reax[7]
variable ehb equal c_reax[8]
variable et equal c_reax[9]
variable eco equal c_reax[10]
variable ew equal c_reax[11]
variable ep equal c_reax[12]
variable efi equal c_reax[13]
variable eqeq equal c_reax[14]

#variables
variable T equal 300.0
variable Dt equal 200
variable dt equal 0.5
variable dp equal ${Dt}/${dt}
variable ttotal equal 400000
```

```
variable Ntotal equal 1000*${ttotal}/${dt}
variable stps equal step
variable time equal v_stps*${dt}
variable kz equal 0.02877697842
variable kxy equal 100000.0
variable vz equal 0.000001
variable atm_ref1 equal 1
variable atm_ref2 equal 2

#set group of atoms
group tip id 87:100
group surf id 101:114

#charge equilibration
fix qeqreax all qeq/reax 1 0.0 10.0 1.0e-6 reax/c

#fix linear and angular momentum
fix mom_tip tip momentum 1 linear 1 1 1 angular
fix mom_surf surf momentum 1 linear 1 1 1 angular

#initial centers of mass
compute cm_surf surf com
variable 0zcm_tip equal 7.0047471429
variable 0ycm_tip equal 0.0146650000
variable 0xcm_tip equal -0.0021085714
variable 0zcm_surf equal -17.8695671429
variable 0ycm_surf equal 0.0146650000
```

```
variable 0xcm_surf equal 0.0021085714

variable 0zcm_adicional equal 50.0 #0 if the simulation is performed from the begining
# any value to move the cantilever at time = 0

#set virtual springs
fix tipxy tip spring tether ${kxy} ${0xcm_tip} ${0ycm_tip} NULL 0.0
fix surfxy surf spring tether ${kxy} ${0xcm_surf} ${0ycm_surf} NULL 0.0
fix surfz surf spring tether 100000.0 NULL NULL ${0zcm_surf} 0.0

#Minimization
neighbor 0.2 nsq
velocity all create $T 53244 dist gaussian mom no rot no
dump mini1 all xyz 1 minimization.xyz
dump_modify mini1 first yes
minimize 1.0e-8 1.0e-10 10000 100000
undump mini1

#Equilibration
variable eq_fuerza equal ${0zcm_adicional}*${kz}
fix equi1 all nve
fix equi2 all langevin $T $T 50.0 14588
fix push_eq tip addforce 0.0 0.0 v_eq_fuerza o
dump eq_out all xyz ${dp} nvt_equilibration.xyz
run 300000
unfix equi1
unfix equi2
```

```
unfix push_eq
undump eq_out

#Pulling
reset_timestep 0

variable t world 280.0 300.0 330.0 340.0 380.0 400.0 450.0
variable rep world 0 1 2 3 4 5 6

velocity all scale $t
fix temp_1 all nve
fix temp_2 all langevin $t $t 50.0 12345
fix ff tip store/force

#Center of mass
compute AveCantiZ tip reduce ave z
fix avecZ tip ave/time 1 1 1 c_AveCantiZ
variable ZC equal f_avecZ

#Center of mass surf
compute AveCantiZsurf surf reduce ave z
fix avecZsurf surf ave/time 1 1 1 c_AveCantiZsurf
variable ZCsurf equal f_avecZsurf

#distance between reference atoms:
variable xdis equal x[${atm_ref1}]-x[${atm_ref2}]
variable ydis equal y[${atm_ref1}]-y[${atm_ref2}]
```

```
variable zdis equal z[${atm_ref1}]-z[${atm_ref2}]
variable distance equal sqrt(v_xdis*v_xdis+v_ydis*v_ydis+v_zdis*v_zdis)

#define force to apply
variable new_zcm equal ${vz}*v_stps*${dt}+${0zcm_tip}+${0zcm_adicional}
variable delta equal v_new_zcm-v_ZC
variable fuerza equal ${kz}*v_delta
variable dif_zcm equal v_ZC-${0zcm_tip}
variable surf_nCM equal v_new_zcm-v_ZCsurf
fix p1 all print ${dp} "${time} ${surf_nCM} ${distance} ${t} ${new_zcm} ${ZC} ${delta}
${fuerza} ${dif_zcm}" screen no file control.${rep}

#the pushing
fix pushing tip addforce 0.0 0.0 v_fuerza

#Molecular dynamics settings
timestep ${dt}
thermo ${dp}
thermo_style custom step etotal pe ke temp press density

dump eq_out all xyz ${dp} dump.temper.${rep}
temper ${Ntotal} ${dp} $t temp_2 2343 62487
```

6 Example input for the electron transport computations using DFTB+

Source or drain:

Geometry = {

GenFormat {

«< 'gen_newsystem.gen'

}

}

Transport {

Device {

AtomRange = 1 108

}

Contact {

Id = "source or drain"

AtomRange = 109 180

}

Contact {

Id = "drain"

AtomRange = 181 252

}

Task = ContactHamiltonian{

ContactId = source

}

```
}

Hamiltonian = DFTB {
SCC = Yes
SCCTolerance = 1e-7
MaxSCCIterations = 400
Mixer=Broyden{MixingParameter=0.001}
OrbitalResolvedSCC = Yes
MaxAngularMomentum = {
C = "p"
H = "s"
}

Filling = Fermi {
Temperature [Kelvin] = 0
}
SlaterKosterFiles = Type2FileNames{
Prefix = "/slako/mio/mio-1-1/"
Separator = "-"
Suffix = ".skf"
LowerCaseTypeName = No
}
KpointsAndWeights = SupercellFolding{
25 0 0
0 1 0
0 0 1
0.0 0.0 0
```

}

}

Device:

```
Geometry = {
GenFormat {
«< 'gen_newsystem.gen'
}
}

Transport {
Device {
AtomRange = 1 108
}
Contact {
Id = "source"
AtomRange = 109 180
FermiLevel [eV] = # value from source output
Potential [eV] = 0.00
}
Contact {
Id = "drain"
AtomRange = 181 252
FermiLevel [eV] = # value from drain output
Potential [eV] = 0.00
```

```
}
Task = UploadContacts {}

}

Hamiltonian = DFTB {
SCC = Yes
SCCTolerance = 1e-7
MaxSCCIterations = 2000
Mixer=Broyden{MixingParameter=0.01}
OrbitalResolvedSCC = Yes
MaxAngularMomentum = {
C = "p"
H = "s"
}

Filling = Fermi {
Temperature [Kelvin] = 0
}

SlaterKosterFiles = Type2FileNames{
Prefix = "/slako/mio/mio-1-1/"
Separator = "-"
Suffix = ".skf"
LowerCaseTypeName = No
}
```

```
Electrostatics = Poisson {

PoissonBox [Angstrom] = 50.0 50.0 50.0

MinimalGrid [Angstrom] = 0.4 0.4 0.4

AtomDensityTolerance = 1e-5

SavePotential = No

}


Eigensolver = GreensFunction {

LocalCurrents = No

}


}


Analysis {

TunnelingAndDOS {

EnergyRange [eV] = {# desired range}

EnergyStep [eV] = # desired energy step

}

}
```
